# Supplementary material for: Monomeric ephrinB2 binding induces allosteric changes in Nipah virus G that precede its full activation
Source: Nat Commun. 2017 Oct 3;8:781. doi: 10.1038/s41467-017-00863-3 (PMC5626764; doi:10.1038/s41467-017-00863-3)
Supplement: Supplementary file 1 — Supplementary Information [file 41467_2017_863_MOESM1_ESM.pdf]

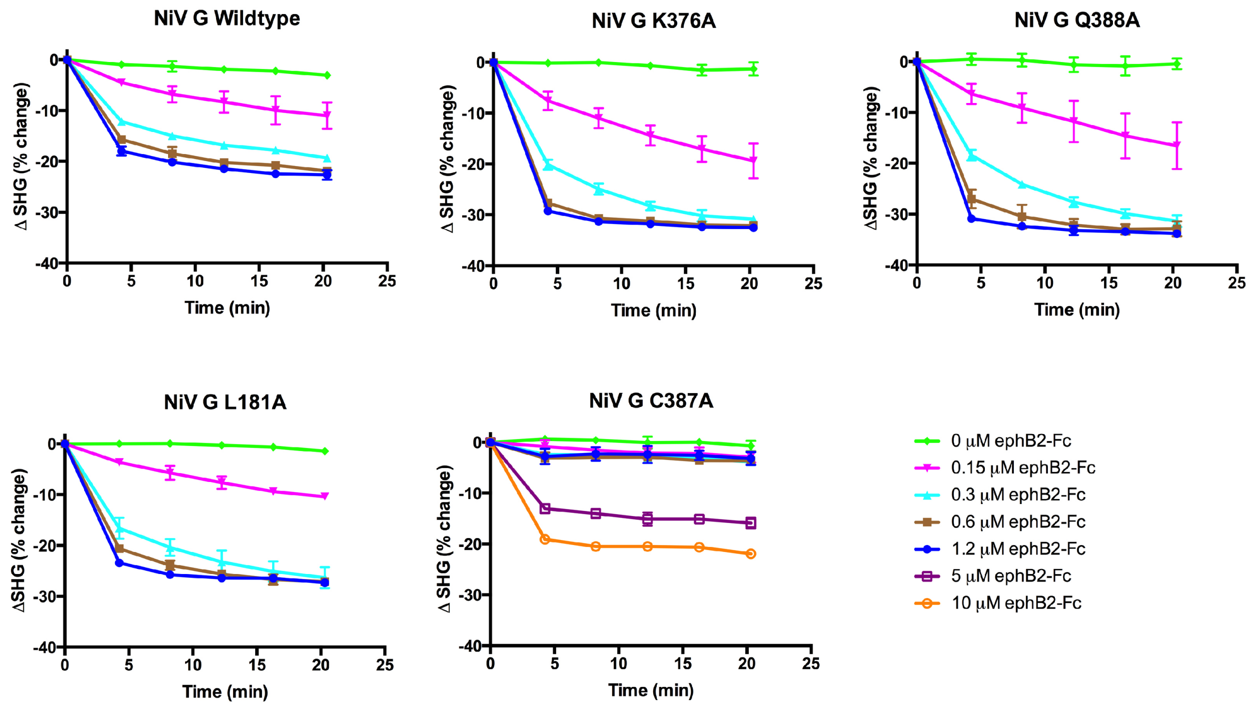

Supplementary Figure 1
SHG kinetic traces for binding of ephrinB2-Fc at varying concentrations to NiV G

NiV G ectodomain constructs were bound to Ni-NTA-lipid containing supported lipid bilayer at 0.5 μM. Mean and s.d. values for SHG data are shown from a representative experiment, n=3.

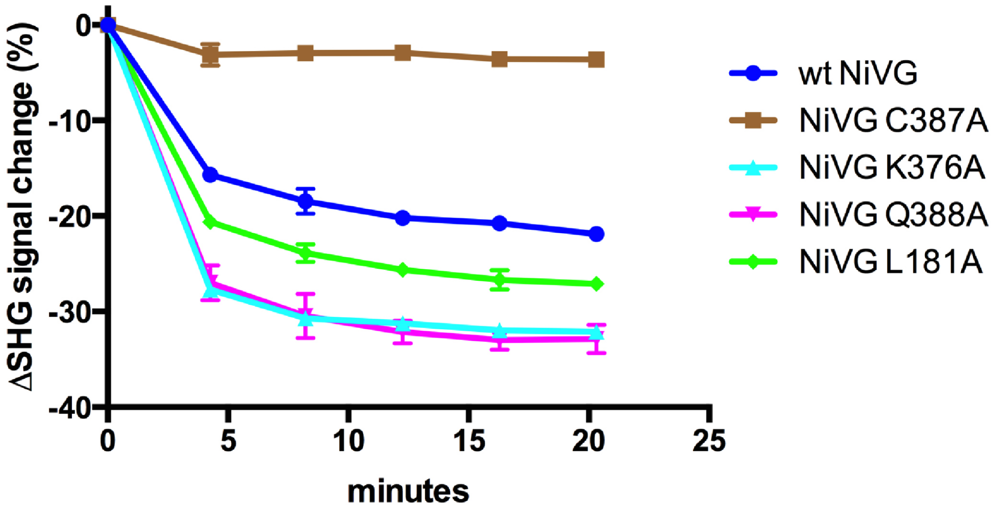

Supplementary Figure 2

SHG kinetic traces for binding of ephrinB2-Fc at 0.6 μM to NiV G ectodomain constructs. Mean and s.d. values for SHG data are shown from a representative experiment, n=3.

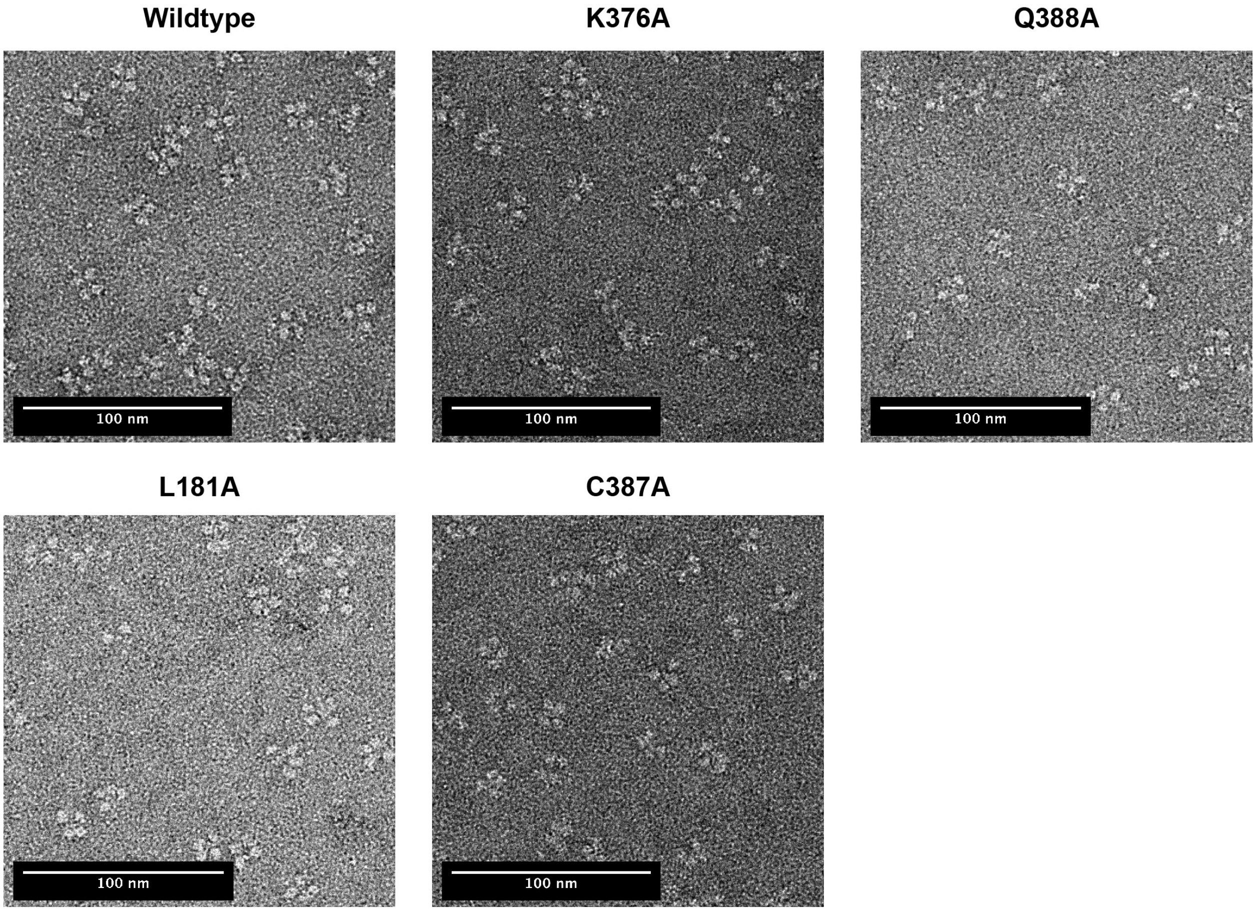

Supplementary Figure 3

Representative raw negative stain electron microscopy images of NiV G ectodomain constructs

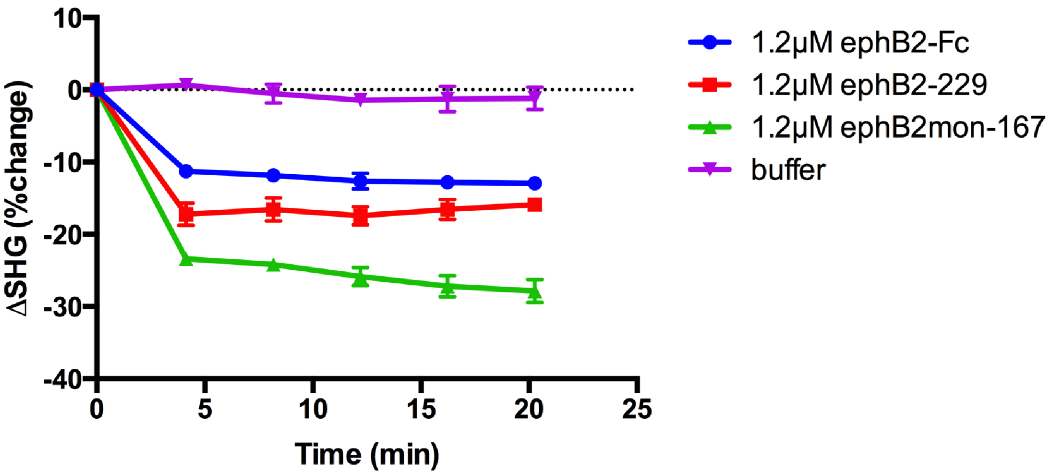

Supplementary Figure 4

SHG kinetic traces for binding of ephrinB2 constructs at 1.2 μM to wildtype NiV G ectodomain
Mean and s.d. values for SHG data are shown from a representative experiment, n=3.

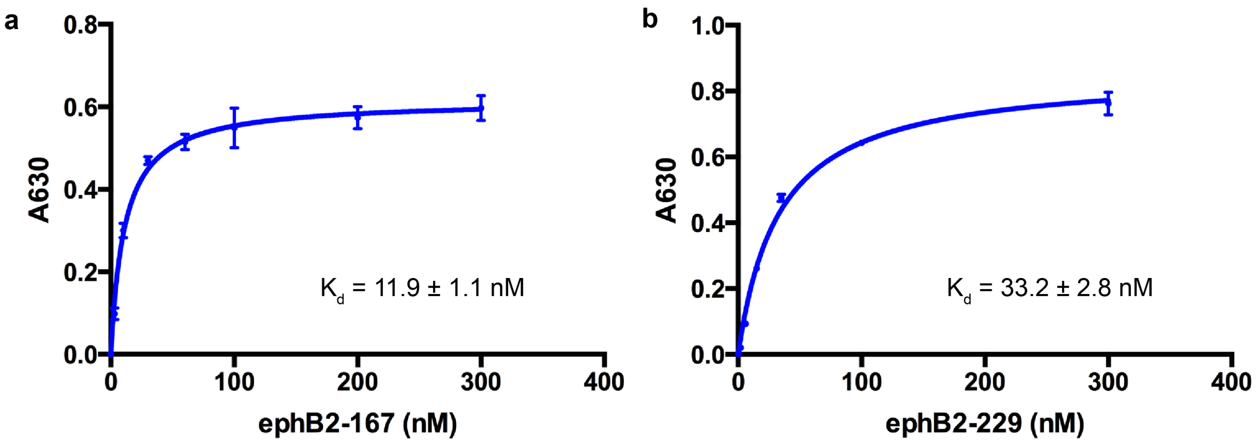

Supplementary Figure 5

Binding affinity of monomeric ephrinB2 constructs to NiV G

a) Binding affinity of ephrinB2-167 to wildtype NiV G ectodomain measured by direct ELISA. Mean and s.d. values for ELISA data are shown from a representative experiment, n=2. b) Binding affinity of ephrinB2-229 to wildtype NiV G ectodomain measured by direct ELISA. Mean and s.d. values for ELISA data are shown from a representative experiment, n=2.

a

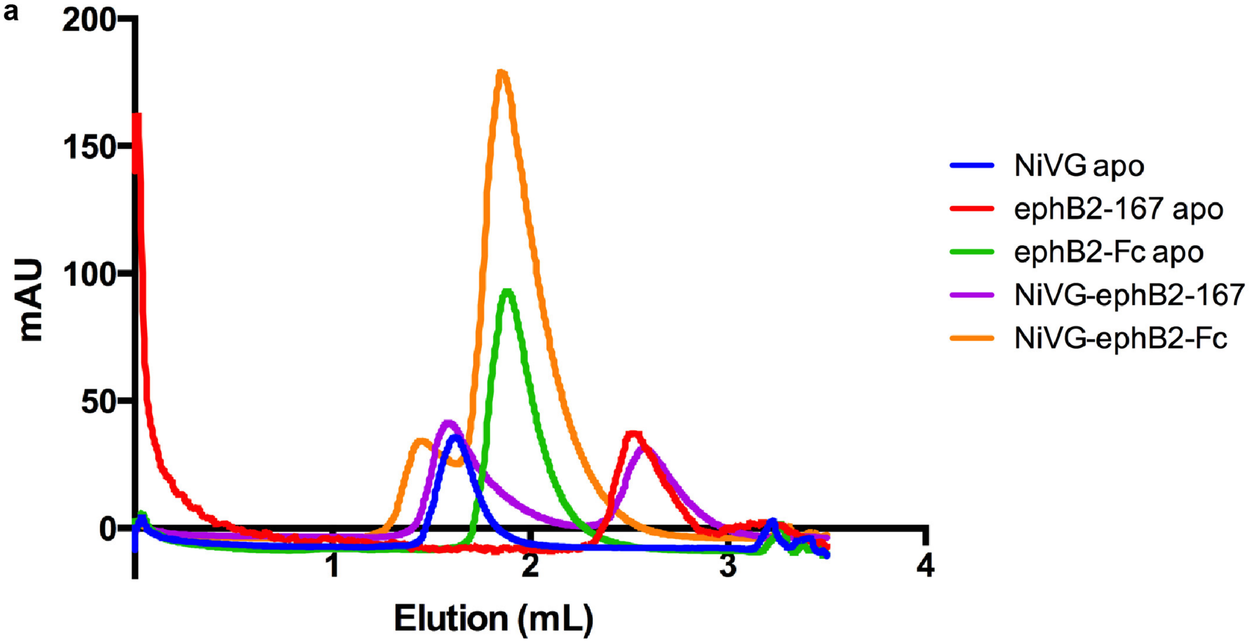

b

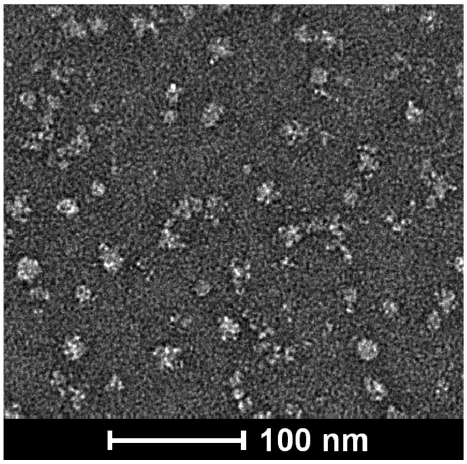

Supplementary Figure 6

Sample preparation of NiV G-ephrinB2 complexes for negative stain EM analysis
a) Size exclusion of NiV G-ephrinB2 complexes with apo-protein controls. Samples were injected at 100 μL injection volumes and run at 0.2 mL/min on a S200 5/150 GL column with 3 mL bed volume. b) Representative raw negative stain particles of NiV G-ephrinB2-Fc complex peak fraction at elution volume 1.4 mL.

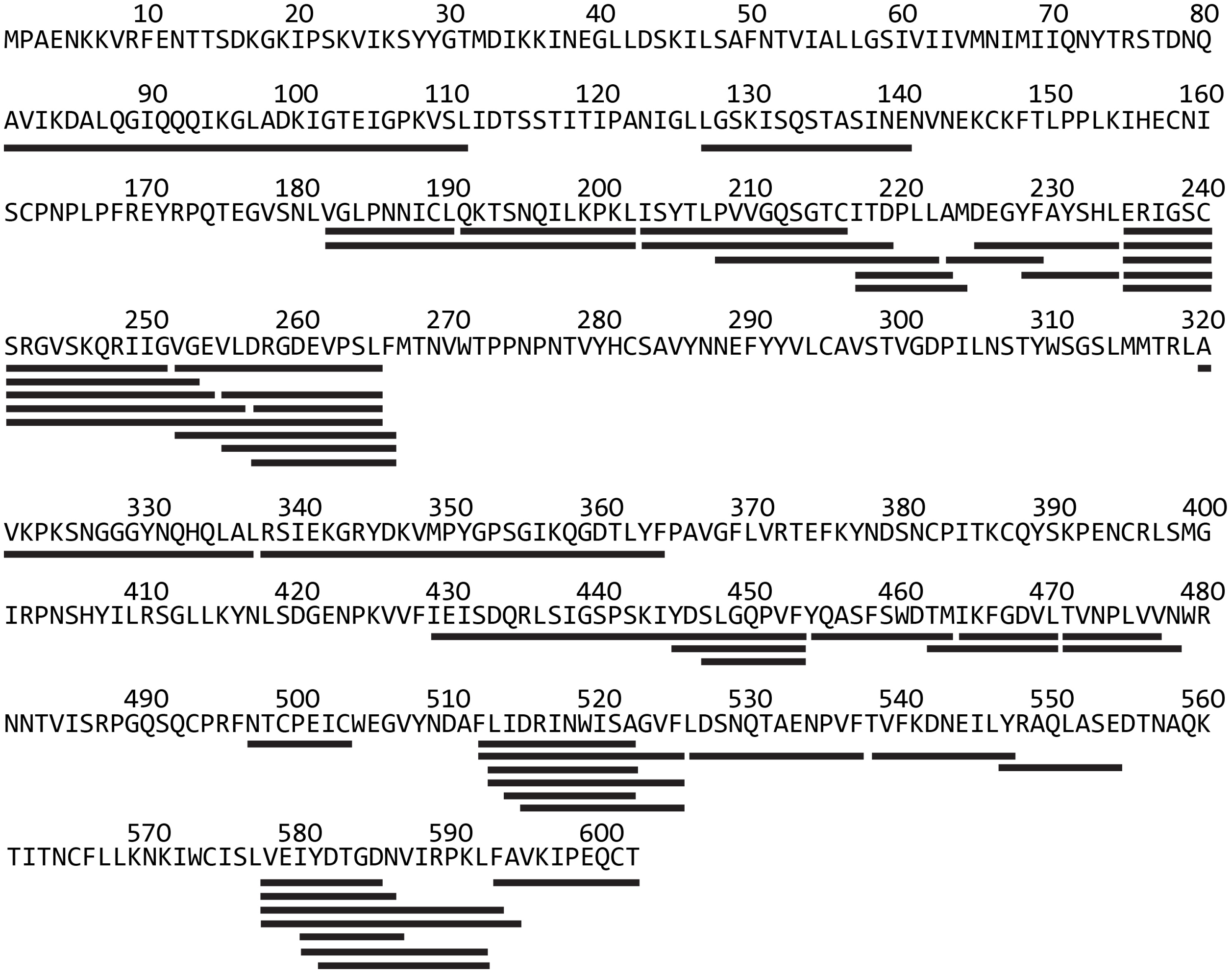

Supplementary Figure 7

Peptide coverage map of NiV G obtained by hydrogen-deuterium exchange mass spectrometry

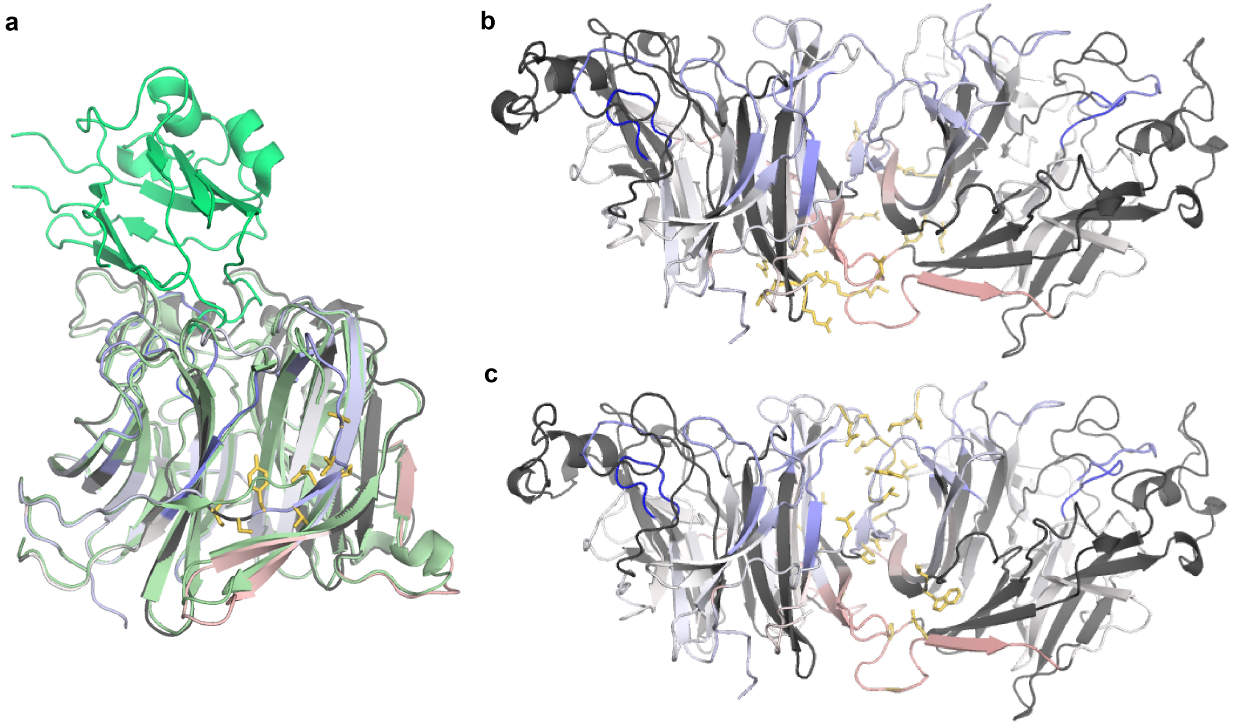

Supplementary Figure 8.

Notable residues from molecular dynamics simulations mapped onto HDX-MS heat maps

a) HDX-MS exchange rates were mapped onto a model of NiV G based on the crystal structure of apo-HeV G (PDB ID 2X9M). NiV G residues 203-211, which undergoes the most conformational change in molecular dynamics simulations (Leighty and Varma, 2013) is shown as yellow side chain sticks. The crystal structure of NiV G bound to ephrinB2 (PDB ID 2VSM) is superposed on the NiV G model. b) NiV G residues that form a dimer interface only when unbound as identified by molecular dynamics simulations (Dutta et. al., 2016) are shown as yellow side chain sticks. HDX-MS exchange rates were mapped onto the a model of NiV G based on the crystal structure of the apo-HeVG dimer (PDB ID 2X9M), with the sequence mutated to that of NiV G. c) NiV G residues that form a dimer interface only when bound by monomeric ephrinB2 as identified by molecular dynamics simulations (Dutta et. al., 2016) are shown as yellow side chain sticks. HDX-MS exchange rates were mapped onto the crystal structure of the apo-HeVG dimer (PDB ID 2X9M), with the sequence mutated to that of NiV G.

Supplementary Table 1. Distribution of SHG dye labels on NiV G ectodomain lysines in wildtype and site-directed mutants obtained by mass spectrometry (%)

	K84	K100	K130	K199	K201	K246	K386	K415	K569 & K571
Wildtype	20.2	5.8	8.8	6.4	27.1	11.4	12.4	7.1	0.3
K376A	16.0	4.7	22.9	4.9	24.1	11.2	1.1	11.2	3.3
C387A	16.2	4.9	16.9	5.9	29.3	11.3	1.0	6.7	6.5
Q388A	17.1	5.0	21.6	4.4	25.3	10.6	0.9	7.2	5.7
L181A	17.6	8.9	28.3	2.1	19.0	11.0	6.7	2.4	4.0

Supplementary Table 2. DNA sequence of NiV G ectodomain construct

ATGCTGTCCGCCATCGTGCTGTACGTGCTGCTGGCCGCCGCCGCCCACAGCGCCTTCGCCCACC
ACCACCACCACCACCCCCCTGACGACGATGATAAACAGAATTACACCCGGAGCACCGACAATCA
GGCCGTGATCAAAGATGCCCTGCAGGGCATTCAGCAGCAGATTAAGGGACTGGCCGACAAAATC
GGGACCGAAATTGGACCCAAGGTGAGCCTGATCGATACCAGCAGCACAATTACAATTCCCGCCA
ATATTGGGCTGCTGGGCTCCAAGATCAGCCAGTCCACCGCCTCCATCAACGAGAACGTGAATGA
AAAATGCAAGTTTACCCTGCCTCCCCTGAAGATCCACGAGTGCAACATCAGCTGCCCCAACCCA
CTGCCCTTCAGAGAGTACCGCCCACAGACCGAGGGAGTGTCCAACCTGGTGGGACTGCCCAACA
ACATTTGCCTGCAGAAAACCTCCAACCAGATTCTGAAGCCTAAACTGATCAGCTACACCCTGCC
AGTGGTGGGCCAGTCCGGCACATGCATCACAGACCCTCTGCTGGCCATGGATGAGGGATATTTC
GCCTACAGCCACCTGGAGCGGATCGGCTCCTGTTCTAGGGGCGTGTCCAAACAGAGAATCATCG
GCGTGGGCGAGGTGCTGGACAGAGGCGATGAAGTGCCTTCCCTGTTCATGACAAACGTGTGGAC
CCCCCCCAATCCAAATACCGTGTACCACTGCTCTGCCGTGTACAACAATGAGTTCTATTATGTG
CTGTGTGCCGTGTCCACAGTGGGAGACCCTATCCTGAATAGCACCTACTGGTCCGGCTCTCTGA
TGATGACACGCCTGGCCGTGAAGCCCAAATCTAACGGGGGGGGCTATAATCAGCACCAGCTGGC
CCTGAGGAGCATCGAAAAGGGCAGGTACGACAAGGTGATGCCCTATGGGCCTAGCGGGATCAAG
CAGGGGGACACCCTGTACTTTCCAGCCGTGGGCTTTCTGGTGCGCACCGAGTTCAAGTACAATG
ACTCCAACTGTCCAATCACCAAGTGCCAGTACTCTAAGCCTGAGAACTGTAGGCTGAGCATGGG
GATCAGACCTAATTCCCACTATATTCTGAGAAGCGGACTGCTGAAGTACAATCTGTCCGATGGG
GAGAACCCCAAGGTGGTGTTTATTGAAATCTCCGATCAGCGGCTGAGCATCGGCAGCCCTAGCA
AGATCTACGACTCTCTGGGCCAGCCAGTGTTCTATCAGGCCTCCTTCTCCTGGGATACCATGAT
TAAGTTCGGCGACGTGCTGACCGTGAATCCACTGGTGGTGAACTGGAGAAACAACACAGTGATT
AGCCGCCCAGGCCAGTCCCAGTGTCCACGGTTTAACACCTGTCCAGAGATTTGTTGGGAGGGGG
TGTACAATGACGCCTTCCTGATTGATCGCATCAATTGGATCAGCGCCGGGGTGTTCCTGGATTC
CAACCAGACCGCCGAAAATCCTGTGTTCACCGTGTTCAAAGATAACGAGATTCTGTACAGAGCC
CAGCTGGCCAGCGAGGACACCAACGCCCAGAAGACCATCACAAACTGTTTTCTGCTGAAGAATA
AGATCTGGTGTATCAGCCTGGTGGAGATCTACGACACCGGAGATAACGTGATTAGACCAAAGCT
GTTCGCCGTGAAGATTCCCGAGCAGTGCACCTAA

Supplementary Table 3. Primers

Primer	Sequence (5'-3')
NiVG_Gibson_For	AGCAGGCTTTAAAGGAACCAATTCAGTCGACACCATGCTGTCCGCCATCGTGCTG
NiVG_Gibson_Rev	AAGCTGGGTCTAGATATCTCGAGTGCGGCCGCTCATTAGGTGCACTGCTCGGGAAT
NiVG_K376A_For	GCACCGAGTTCGCATACAATGAC
NiVG_K376A_Rev	GTCATTGTATGCGAACTCGGTGC
NiVG_C387A_For	CAATCACCAAGGCCCAGTACTC
NiVG_C387A_Rev	GAGTACTGGGCCTTGGTGATTG
NiVG_Q388A_For	CCAAGTGCGCGTACTCTAAGC
NiVG_Q388A_Rev	GCTTAGAGTACGCGCACTTGG
NiVG_L181A_For	GTGTCCAACGCGGTGGGACT
NiVG_L181A_Rev	AGTCCCACCGCGTTGGACAC
EphB2mon_Stag_For	TTCGAGCGGCAGCACATGGATTCTCCCCCTGACGACGACGAC
EphB2mon_Stag_Rev	CTTGGCAGCGGCGGTTTCTTTGGCGAAGGCAGAGTGGGCGGC
EphB2_168-229_For	GATGAAAGTGGGCCAGGACGCAAGCTCTGCTGGGTCC
EphB2_168-229_Rev	CTCGAGCGGCCGCTTATTAGGCAAACAAAGCCACT
EphB2_167_Rev	GTCCTGGCCCACTTTCATCAGG
pTT5_EcoRI_For	CCCAGGTCCAAGTTTAAACGGATCTCTAGCGAATTC
